# Pneumococcal meningoencephalitis: evolutive particularities in a case with leukemia. Case report

**DOI:** 10.1186/1471-2334-14-S7-P94

**Published:** 2014-10-15

**Authors:** Ioana-Raluca Todoran, Cristina Gîrbovan, Carmen Chiriac, Brînduşa țilea, Anca Georgescu, Andreea Bodea-Alucăi

**Affiliations:** 1 Clinic of Infectious Diseases I, Tîrgu Mureş, Romania; 2 University of Medicine and Pharmacy Tîrgu Mureş, Romania

## Background

*Streptococcus pneumoniae* represents the main cause of meningoencephalitis in adults. Penicillin-resistant *Streptococcus pneumoniae* has a high prevalence, representing an important cause of mortality and morbidity.

## Case report

We present the case of a female patient, aged 62 years, diagnosed with chronic lymphatic leukemia and *Listeria monocytogenes* meningitis in the past two years, who was hospitalized in the Clinic of Infectious Diseases I with symptoms of high grade fever, altered state of consciousness, generalized tonic-clonic seizures, right oculocephalogyric deviation and coma, Glasgow Coma Scale 3 when admitted, following intense care unit and ventilatory support for ten days.

After two days of hospitalization the patient developed a labial hemorrhagic necrotic herpetic lesion. Lumbar puncture with cerebrospinal fluid assessment revealed a turbid fluid with leukocyte count 620/3 cells/μL, glucose 19 mg/dL, Pandy reaction was positive. Blood count interpretation indicated leukocytosis 14,000 cells/cmm, lymphocytosis 70% and neutropenia 23%. Cerebrospinal fluid Gram strains reveals Gram-positive diplococci and CSF culture grew penicillin-resistant *Streptococcus pneumoniae*. Computer tomography of the brain was negative for an acute intracranial pathology, and showed fluid collections and mucosa thickening of maxillary sinus.

Treatment was promptly administered with meropenem, vancomycin, corticosteroids, acyclovir and intensive care support led to patient survival, with a long, but favorable evolution of three weeks.

## Conclusion

Pneumococcal meningoencephalitis is a severe disease with high incidence in immunocompromised patients, which can lead to systemic complications in patients aged 60 years and older. A promptly administered treatment with broad-spectrum antibiotics is necessary, since resistance to penicillin of *Streptococcus pneumoniae* is common. Clinical evolution of patient was favorable, despite the multiple comorbidities and the profoundly altered general condition on admission and a low score on the Glasgow coma scale.

## Consent

Written informed consent was obtained from the patient for publication of this Case report and any accompanying images. A copy of the written consent is available for review by the Editor of this journal.

